# The Open Access Malaria Box: A Drug Discovery Catalyst for Neglected Diseases

**DOI:** 10.1371/journal.pone.0062906

**Published:** 2013-06-17

**Authors:** Thomas Spangenberg, Jeremy N. Burrows, Paul Kowalczyk, Simon McDonald, Timothy N. C. Wells, Paul Willis

**Affiliations:** 1 Medicines for Malaria Venture (MMV), Geneva, Switzerland; 2 SCYNEXIS Inc., Research Triangle Park, North Carolina, United States of America; National University of Singapore, Singapore

## Abstract

Historically, one of the key problems in neglected disease drug discovery has been identifying new and interesting chemotypes. Phenotypic screening of the malaria parasite, *Plasmodium falciparum* has yielded almost 30,000 submicromolar hits in recent years. To make this collection more accessible, a collection of 400 chemotypes has been assembled, termed the Malaria Box. Half of these compounds were selected based on their drug-like properties and the others as molecular probes. These can now be requested as a pharmacological test set by malaria biologists, but importantly by groups working on related parasites, as part of a program to make both data and compounds readily available. In this paper, the analysis and selection methodology and characteristics of the compounds are described.

## Introduction

Approximately 260 million people are affected each year by malaria, with around 655,000 deaths. Children under 5 years of age and pregnant women are particularly affected [1]. The disease is widespread in sub-Saharan Africa where the economic and humanitarian burden is considerable. Malaria is caused by parasites of the genus *Plasmodium* with *Plasmodium falciparum* and *Plasmodium vivax* being predominantly responsible for the mortality and morbidity, respectively. The current gold standard treatments for malaria are the artemisinin combination therapies: combinations of derivatives of the natural product artemisinin, and aminoquinolines or aminoalcohols, the descendants of quinine [2]–[4]. Five such artemisinin combination therapies have been approved by either stringent regulatory authorities or the World Health Organization's prequalification department. A dispersible fixed dose combination of artemether and lumefantrine specifically designed for children, Coartem®-*Dispersible* was developed by a collaboration of Medicines for Malaria Venture (MMV) [Medicines for Malaria Venture (MMV) is a not for profit public private partnership whose focus is on the discovery, development and launch of small molecule anti-malarial agents. MMV raises and distributes funds working with many collaborators around the world.] and Novartis. Since the launch in 2009 over 150 million treatments of this life-saving medicine had been delivered to 35 malaria-endemic countries. However, there is continually concern that *P. falciparum* strains with decreased speed of parasite killing are present in the border regions of Cambodia, Thailand and Myanmar [5]–[6]. This is putting increased pressure on the partner medication, and highlighting an urgent need for the development of new anti-malarial medicines over the next decade [1],[4]. To further support the malaria eradication agenda new drugs with transmission blocking or liver stage activity are also required [7]–[10].

Since 2008, almost six million compounds have been screened against the blood stages of *Plasmodium falciparum*. Approximately 0.5% of these compounds showed activity consistent with an EC_50_ of less than one micromolar. The majority of these compounds have been made available to the scientific community through publications from consortia led by GlaxoSmithKline (GSK), Novartis, and St. Jude Children's Research Hospital, Memphis (St Jude) [11]–[13]. These publications underline the power of phenotype screens in identifying new scaffolds or chemotypes with promising activity [14]. Historically, organizations have not published the full hit sets from High Throughput Screens (HTS). The decision to publish such a large set of early screening data (20,000 compounds) may revolutionize the drug development paradigm by allowing any group to initiate a drug discovery project, based on their own analysis of the results. Further research could lead off in several directions. First, new projects could be formed to identify the biological pathway or mechanism of action of the compounds against *Plasmodium*, opening up new classes of pharmacologically validated chemotypes and targets. This includes testing against the different stages of the parasite lifecycle [15]. Second, these compounds may be useful for starting hit-to-lead campaigns, provided they are judged to have medicinal chemistry potential with suitable pharmacokinetic and metabolic properties. Third, it is possible that they could serve as a starting point for finding new compounds which are capable of killing other parasites or pathogens. Since parasites can share common biological pathways, antimalarial leads will provide useful drug discovery starting points in other infectious diseases allowing a better understanding of the commonalities required in anti-infective compounds, which will facilitate breaking down the silos of diseases which have often been considered separately.

However, all of these approaches are in some ways hampered by two issues. In most cases it is a prerequisite to be able to obtain physical samples of the chemical compounds for further study, and the groups responsible for screening did not originally plan to provide these molecules. In addition, many of the biological systems in which these compounds would be tested are not suitable for testing such large numbers of compounds. There needs to therefore be some simplification of the collection. To overcome these barriers, a diverse collection of anti-malarial compounds has been designed and assembled. The methodology and principles underpinning the selection of these compounds for the Malaria Box are discussed in detail. It is important to stress that the primary selection criteria was the commercial availability of compounds. The final “Open Access Malaria Box” has been produced and delivered, with the overarching aim of catalyzing research towards the discovery of new efficacious small molecules suitable for clinical development. The Malaria Box is free of charge. All that is asked in return is that any data gleaned from research on the Malaria Box is placed into the public domain and ideally published.

## Materials and Methods

The data reported by GSK [11], Novartis [12], St. Jude Children's Research Hospital [13] constitute the initial set of compounds used in this effort. These data, including structures and inhibitory activity against *P. falciparum* 3D7, are available for download from the ChEMBL-NTD database (EBI website. Available: http://www.ebi.ac.uk/chemblntd, last accessed on 2013 April 18.). The St. Jude's dataset includes 1536 compounds; the Novartis dataset includes 5708 compounds made available for public disclosure, representing about half of their screening hits; and the GSK dataset includes all 13519 compounds identified from their screening programme.

### Dataset Preparation

Prior to any analysis, compounds in each dataset were processed to (1) strip salts, (2) remove small fragments, (3) deprotonate bases/protonate acids, (4) generate canonical tautomers, and (5) remove duplicates. This dataset preparation was performed using Pipeline Pilot 8.5 (Accelrys, San Diego, CA). At this point any compound with molecular weight >1000 or any compound with greater than 20 rotatable bonds was removed from further consideration. Following these steps, the St Jude's dataset included 1523 unique structures; the Novartis dataset included 5661 unique structures; and the GSK dataset included 13257 unique structures.

**Figure 1:**
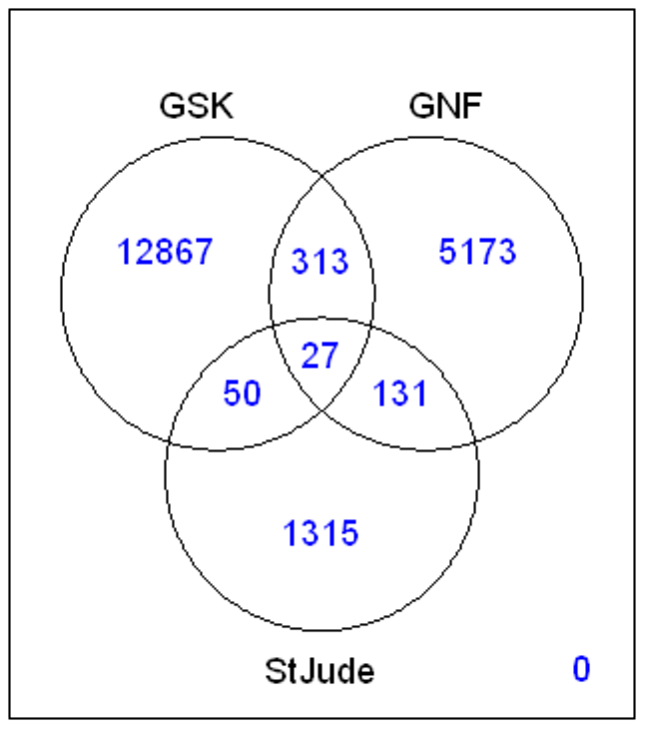
Venn diagram presenting the overlap of structures in the St. Jude, Novartis and GSK datasets. The data was generated using Pipeline Pilot 8.5, and displayed using R 2.14.1.

The canonical SMILES representations of molecules in the St Jude's, GSK and Novartis datasets were compared to identify *inter*-dataset structure duplicates. These results are presented graphically in Figure 1. There are 1315 structures unique to the St Jude's dataset; 5173 structures unique to the Novartis dataset; and 12867 structures unique to the GSK dataset. The St Jude's and Novartis datasets share 158 structures, in common; the Novartis and GSK datasets share 340 structures, in common; and the GSK and St Jude's datasets share 77 structures, in common. There are 27 structures common to all three datasets. There are 19876 structurally unique compounds present in the St Jude's, Novartis and GSK datasets. These structurally unique compounds constitute the compound pool for the selection of a representative subset of antimalarials (*i.e.*, the Malaria Box).

### Dataset Analysis

**Figure 2:**
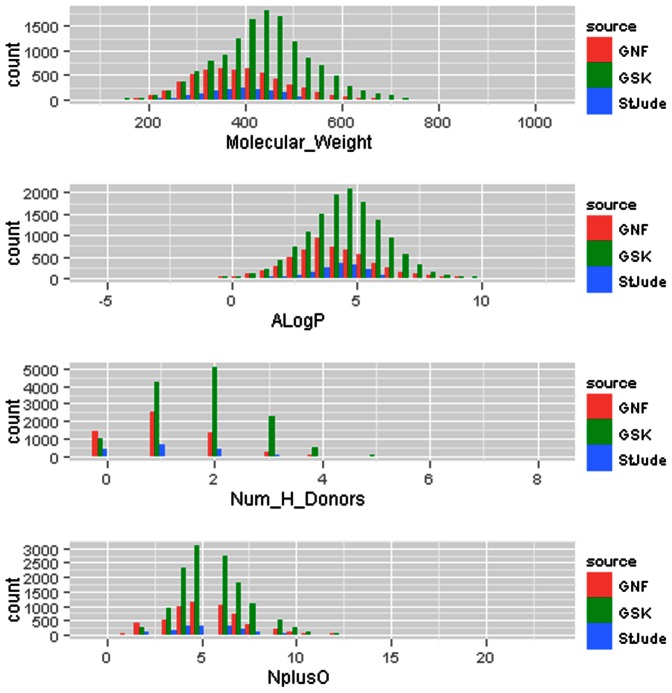
St Jude's, Novartis and GSK datasets profiled with respect to molecular weight, the number of hydrogen-bond donors, ALogP and N+O (nitrogen count plus oxygen count).

Each of the St Jude's, Novartis and GSK datasets was profiled with respect to molecular weight, the number of hydrogen-bond donors, ALogP and N+O (nitrogen count plus oxygen count). These are the four physicochemical quantities that have been used to profile molecules with regards to the likelihood of their becoming successful oral drugs [4]. The results are presented graphically in Figure 2.

## Results

### Chemical diversity of the dataset

**Figure 3:**
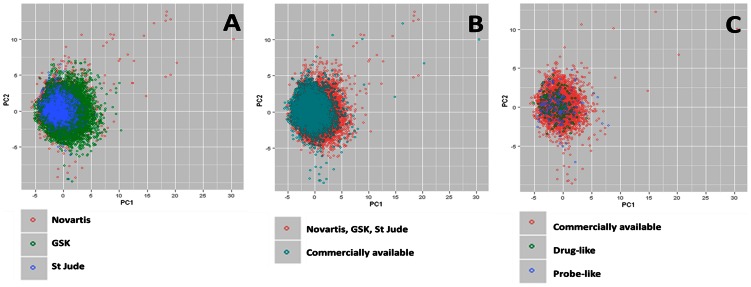
Principal Component Analysis plots. Chemical diversity of the GSK, Novartis and St Jude libraries displayed (Panel A); Overlap in chemical diversity of the combined datasets and the commercially available compounds (Panel B); Overlap in chemical diversity of the commercially available compounds where the drug-like and probe-like chemotypes were annotated (Panel C).

Given the limitations on the number of compounds that can be tested in detail, to maximize the potential impact of the Malaria Box it was important to maximize the structural diversity in the compounds selected [16]. A two dimensional Principal Component Analysis (2D-PCA) was used to assess the chemical diversity of the 20,424 hits that originated from the original screening libraries. The three collections of hits occupy similar property space, with the GSK set showing the greatest diversity, although this is most likely a consequence of it being the largest data set [Figure 3, panel A]. To avoid the cost of resynthesis and to make the compounds readily available for follow-up experiments, the next step consisted in selecting commercially available compounds. Around a quarter (5034) of the hits were accessible through on-line vendors [This search was performed through the eMolecules website found at www.emolecules.com. Last accessed 2013 April 18.]. Subjecting these 5,034 compounds to the same principal component analysis resulted in a good overlap with the initial dataset. This suggests that commercially available compounds are representative of the diversity of the overall set [Figure 3, panel B]. Finally 200 drug-like and 200 probe-like compounds covering the chemical diversity of the commercial set were selected for the Malaria Box [Figure 3, panel C] in the public domain.

### Drug-like and probe-like compounds

**Figure 4:**
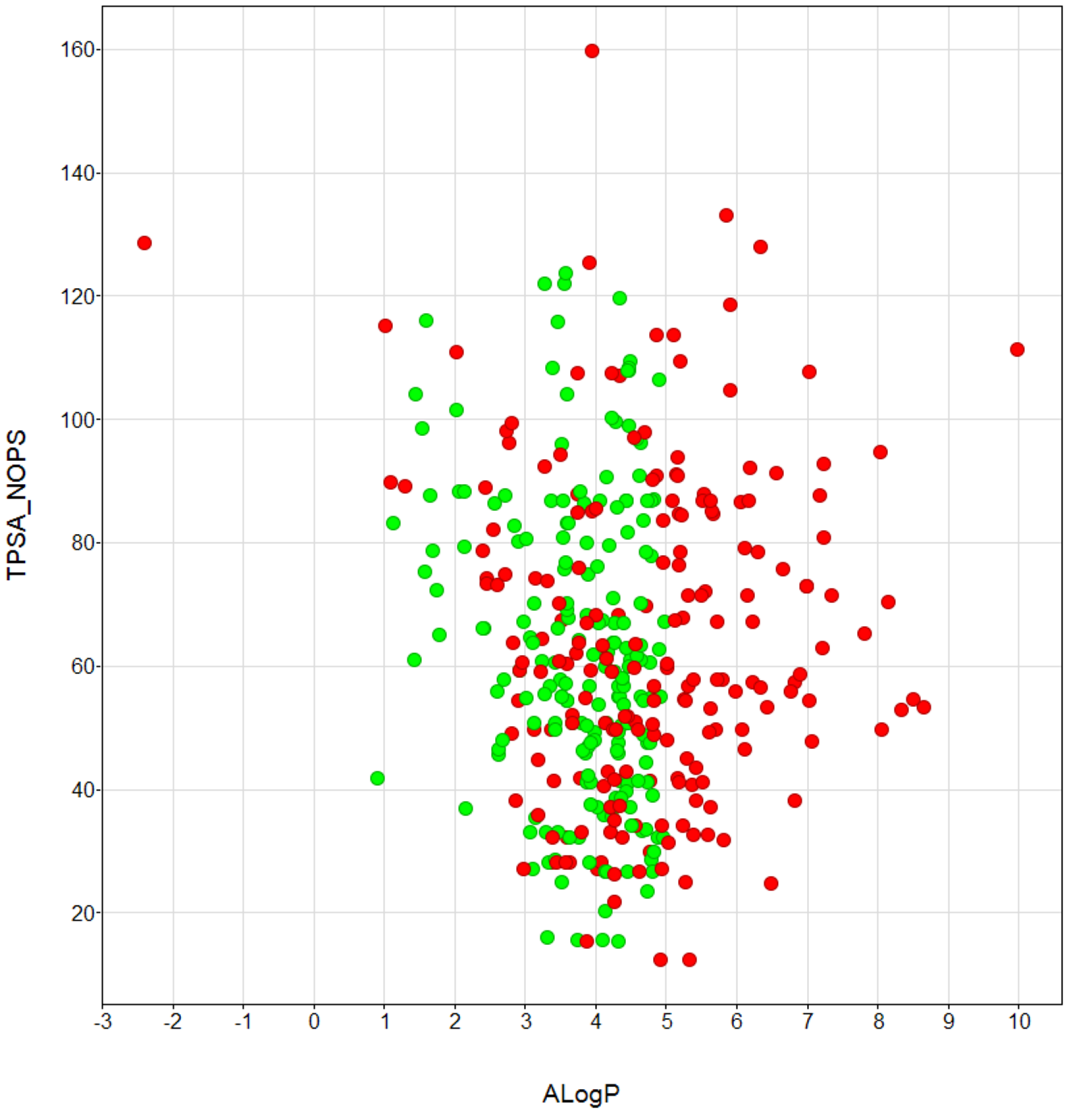
Topological Polar Surface Area versus the ALogP for the Malaria Box drug-like set (green) and probe-like set (red). Displayed with Vortex (v2012.11.17233) Dotmatics Limited 2007, 2012.

The drug-like compound set was chosen from those hits which have rule-of-5-compliant physicochemical properties, often considered a rule of thumb for compounds likely to show acceptable oral absorption [Figure 2 & 4] [17]. Substructure filters were first applied to the 5,034 commercially available compounds to remove known toxicophores [18]–[23]. The remaining structures were further reviewed for liabilities using the **REOS** (Rapid Elimination Of Swill) and the **PAINS** (Pan Assay Interference Compounds) filters [24],[25]. Any compound that failed one or more of these filters was eliminated from the drug-like set and assigned to the probe-like category.

**Figure 5:**
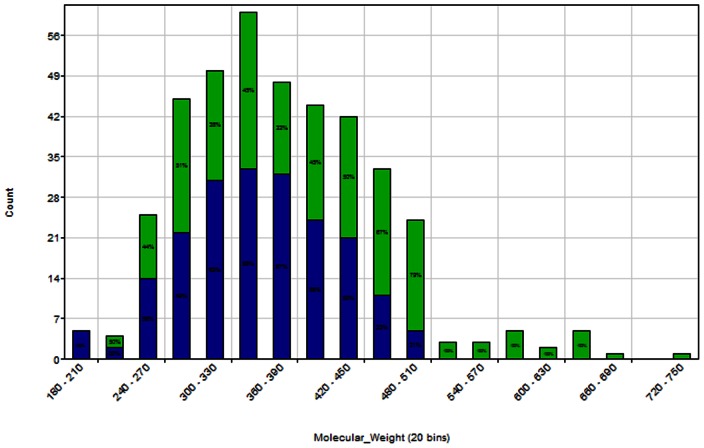
Molecular weight distribution for the drug-like set (blue) and probe-like set (green). Displayed with Vortex (v2012.11.17233) Dotmatics Limited 2007, 2012.

Of note is the molecular weight distribution of the drug-like set: 21.5% are below 300 g/mol, 53% range between 300 and 400 g/mol while only 25.5% are in the range of 400 and 500 g/mol [Figure 5]. This provides space for medicinal chemists when starting their hit-to-lead activities. Conversely the probe-like set displays a different profile: 18% are below 300 g/mol, 35% are comprised between 300 and almost half of this set (47%) has a molecular weight greater than 400 g/mol. Since no deliberate restrictions are applied, the probe-like set represents the broadest cross-section of structural diversity and might find most use as tools for probing biological mechanisms.

From the 5034 compounds that are commercially available, 2693 fitted with the definition of drug-like molecule, leaving the remaining 2341 compounds in the probe-like category.

### Clustering and optimizing the balance between potency and chemical diversity balance

**Figure 6:**
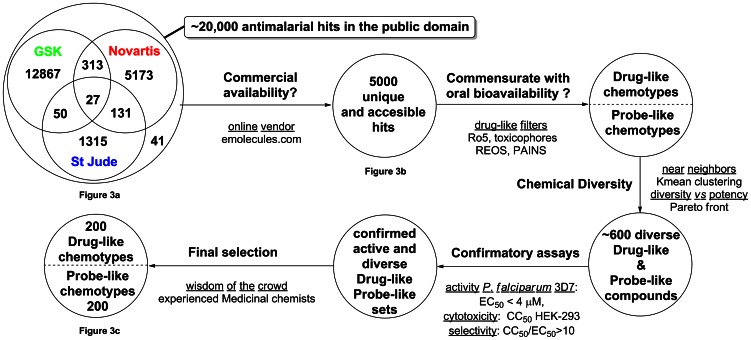
Selection process for the Malaria Box.

To narrow down the number of antimalarial compounds to a manageable representative set, the drug-like and probe-like sets were independently clustered through a Tanimoto-Rogers protocol to furnish 300 clusters in each set allowing no singletons to remain [26]. The Pareto protocol [Deb, K., Agarwal, S., Pratap, A., Meyarivan, T. A Fast Elitist Non-Dominated Sorting Genetic Algorithm for Multi-Objective Optimization: NSGA-II. KanGAL report 200001, Indian Institute of Technology (2000). Online at the CiteSeerX website: http://citeseer.ist.psu.edu/309793.html, last accessed 2013 April 18.] was used to give the best balance between potency and chemical diversity in each of the two subsets. While maximizing the chemical diversity, where possible, inclusion of near neighbours or matched molecular pairs along with significant difference in antiplasmodial activity was attempted. Anticipating that the *in vitro* retesting of a new sample of each compound could lead to a high attrition rate, *ca* 300 drug-like and *ca* 300 probe-like compounds were picked at this stage and used to assemble the confirmatory set [Figure 6].

### Confirmatory compound set

**Figure 7:**
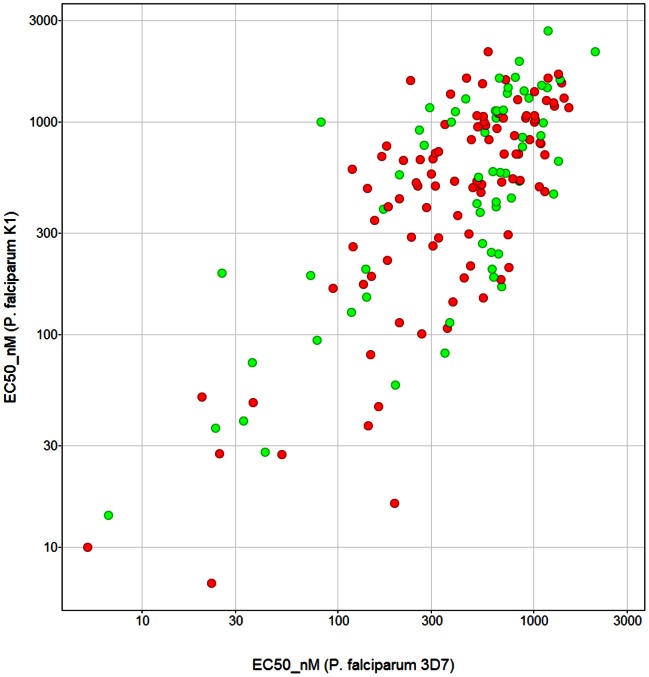
Correlation (Log scale) between the *P. falciparum* 3D7 and K1 EC_50_s for the Malaria Box compounds resulting from the confirmatory screen. Displayed with Vortex (v2012.11.17233) Dotmatics Limited 2007, 2012.

To allow the final selection of the 400 compounds, all ∼600 compounds in the confirmatory set were tested against the *P. falciparum* 3D7 and K1 strains using a DAPI (4′-6-diamidino-2-phenylindole) stain and a fluorescent high content imaging as recently disclosed by Duffy & Avery [27]. Noteworthy, a correlation (Log scale) in activity (EC_50_s) between the *P. falciparum* 3D7 and K1 strains for the Malaria Box compounds was observed (Figure 7). For consideration for inclusion in the Malaria Box a compound had to show an activity of at least 4 µM against *P. falciparum* 3D7, together with a selectivity ratio of at least a 10-fold over the cytotoxicity assay (CC_50_) which was measured using the HEK-293 cell lines. Of the 685 compounds tested, 459 showed an EC_50_<4 µM, which underscores the inherent accuracy of the original screening data. A final collection of 400 were selected by a ‘wisdom of the crowd’ approach using experienced medicinal chemists [28].

### Wisdom of the crowd

From the remaining compounds, a group of experienced medicinal chemists first selected 200 compounds from the drug-like confirmatory set. Then from the remaining compounds, 200 diverse compounds were selected and assigned to the probe-like set. Importantly, some of the compounds in the probe-like set may therefore also conform to Lipinski's rules and be suitable starting points for drug discovery programs.

To make sure that the collection was future-looking, the presence of compounds related to existing chemical scaffolds such as aminoquinolines and endoperoxides as well as prosecuted scaffolds was minimized [13],[29]. Also ∼10% of the Malaria Box is comprised of MMV proprietary compounds.

Compounds for inclusion in the Malaria Box were chosen from the subset of screening hits that were commercially available in sufficient quantities (approximately 5000 of the 20,000 screening hits). This requirement did limit the options for selecting drug-like compounds, but it was possible to select a set of compounds that matched drug-like criteria, as defined by Lipinski's rules [Tables S1&S2].

### Composition and layout of the Malaria Box

No marketed antimalarials were included in the Malaria Box, however, on each plate 16 wells are left empty to allow the addition of the appropriate positive and negative controls. The plate layout of the Malaria Box has been designed to be flexible and to accommodate differing screening capacities. It is available as either 10 µL or 20 µL of a 10 mM DMSO solution, supplied in V-shaped 96-well plates and shipped frozen. Plate A contains the most potent compounds from 40 drug-like and 40 probe-like chemotypes, while plates B-C contain the 160 remaining drug-like compounds and plates D-E contain the 160 remaining probe-like compounds. Groups with very limited screening capacity should therefore order plate A. These sets are available upon request and free of charge from www.mmv.org/malariabox. Full data on the Malaria Box with original GSK/St Jude/Novartis compound number, structure, canonical SMILES, biological data, and select *in silico* physicochemical parameters is available [in [Supplementary-material pone.0062906.s001]] as well as on the MMV website (http://www.mmv.org/research-development/malaria-box-supporting-information). Also detailed, are non-confidential data highlighting whether a particular chemotype/series has already been explored.

## Discussion

We have described the selection, assembly, testing and disposition of 400 diverse drug-like and probe-like confirmed blood-stage active antimalarial compounds. The initial aim of the Malaria Box was to catalyze research into malaria and the discovery of new antimalarial clinical candidates. However, the utility of the Malaria Box goes beyond the malaria field since these biologically active, cell permeable compounds are highly likely to be active against other parasitic or neglected diseases [30],[31]. The overlap between the biologies of the basic apicocomplexan parasites suggests that hits from malaria screens would be useful against leishmaniasis and trypanosomiasis, and even on helminth targets [30]. The new generation of hits from parasitological screens, has now led to the identification of pharmacologically validated targets, many of which are known to be represented in a wide variety of different parasites, and even have homologs in mammalian cells. The fact that to kill a parasite the compounds have to cross membranes and have biological actions, means they may also have activity against mammalian cells, and have applications even in disease areas such as oncology [32]. Ultimately, data generated from the Malaria Box should provide a valuable resource to allow the community to better understand similarities and differences between various parasitic and orphan diseases [33].

There are several notable strengths to the Malaria Box. The antimalarial activity spans a range of EC_50_ values from 30 nM through to 4 µM on a drug sensitive and resistant *P. falciparum* strain. The fact that the box does not contain compounds that are highly optimized to inhibit *P. falciparum* may increase the chances of detecting activity against other diseases. Secondly, where possible, near neighbors were included in the Malaria Box so that on initial screening a first insight in structure activity relationship (SAR) or matched molecular pairs could be obtained [34].

Beyond the scientific aspects of the project, there is also the aspect of increasing access to information and molecules. Molecular biology was transformed in the early days of the human genome project by the availability of expressed sequence tag hits, which were put on public domain databases many years before the human genome was completed. The open access culture of expressed sequence tags underscored the power of having high quality data available to a wide network of end-users. The fundamental difference in the case of chemoinformatics is that unlike with nucleic acid sequences, it is currently not possible to make small molecules to order at a price which is affordable by most biological laboratories. There is therefore a need to provide physical samples of the material to groups that request them. Open source of course means that the authors encourage everyone using this compound deck to place their results in the public domain as soon as possible. Although, it should be emphasized that almost by definition in an open source project the dilemma is that the end-user cannot be forced to disclose their data. The hope here is that groups who identify interesting new activities will consider their discoveries more as intellectual property responsibilities rather than intellectual property rights. As such the discoverer has a responsibility to either explore their discovery and progress the project to the ultimate goal of new therapeutics for mankind, or else return their ideas to the public domain for others to build on. No disease area can afford the luxury of idle data, and especially not in the area of neglected disease. To help continue the virtuous cycle of research, MMV and the European Bioinformatics Institute have established a one-stop-shop for deposition [Upon request, support is provided to upload the data in ChEMBL-malaria] of data generated on the Malaria Box [35],[36]. The new resource consolidates publicly available malaria data related to drugs, compounds, targets and assays into an easy-to-search database [EBI website. Available: https://www.ebi.ac.uk/chembl/malaria, last accessed 2013 April 18].

In the first year of the program, over 120 requests for the compound set have been received, with many of them coming from groups working on different infectious agents. If screening hits are obtained, the following evaluation is suggested to assess the initial quality of the hit. First, checking whether the chemotype is known for the particular target/organism. Second, prioritizing the hits based on the new information that will have emerged about the drug-like properties. Over the next 12 months, then the *in vitro* metabolism of the Malaria Box will be analyzed in detail, and also some of the compounds will be tested for preliminary pharmacokinetics *in vivo*. MMV will report this data. Third, confirming that the compounds are active against a variety of primary or field isolated strains. Fourth, to check through the original screening data and Malaria Box to see whether there are near neighbors which have activity, and then finally expanding the mammalian cell based screens to determine if there is any other limiting cellular toxicology. The subsequent steps could involve the purchase of a fresh solid sample, together with some commercially available near neighbors (Tanimoto-Rogers distance ∼0.85) to validate the initial hit and have a first insight into the structure activity relationships in the chemical series.

## Supporting Information

Table S1 **The list of the 400 compounds contained in the Open Access Malaria Box.** The Excel file (columns A to V) contains the information pertaining to each compound:
- **HEOS_COMPOUND_ID** (as an MMV identification number).
- **Batch_No_March2012** (batch number for 1^st^ round of shipments).
- **Batch_No_May2012** (batch number for 2^nd^ round of shipments).
- **Batch_No_April2013** (batch number for 3^rd^ round of shipments).
- **Smiles** (structure of the compound in a Canonical SMILES format).
- **percent_inh at 2 µM** (% inhibition of 3D7 growth at 2 µM concentration of compound).
- **percent_inh at 5 µM** (% inhibition of 3D7 growth at 5 µM concentration of compound).
- **EC50_nM** (against P. falciparum 3D7, as reported by Prof Avery).
- **ChEMBL_NTD_ID** (compound identity as reported in the ChEMBL database).
- **source** (GSK, GNF, StJude or Commercial libraries).
- **CHEMBL EC50 in µM** (against P. falciparum 3D7, as reported in the ChEMBL database).
- **Set** (Drug-like or Probe-like).
- **Ro5_ViolationCount** (violation of Lipinski's Rule of 5, e.g. 1 = 1 violation of Lipinski's rule).
- **NplusO_Count** (sum of Nitrogen and Oxygen atoms).
- **Molecular_Weight** (in g/mol).
- **Num_H_Donors** (sum of hydrogen bond donors as drawn).
- **ALogP** (calculated partition coefficient).
- **Comment** (if applicable).
- **Plate_March2012** (plate assignation of the compound for 1st round of shipments).
- **Well_March2012** (location on the designated plate for 1st round of shipments).
- **Plate_May2012_April2013** (plate assignation of the compound for 2^nd^ and 3^rd^ round of shipments).
- **Well_May2012_April2013** (location on the designated plate for 2^nd^ and 3^rd^ round of shipments). 
(XLS)

Table S2 **List of vendors used to supply compounds for the Malaria Box, including vendor's web address and the number of compounds from each vendor in the Malaria Box (December 2011).**
(XLSX)
